# Central giant cell granuloma formation in an edentulous area in the posterior portion of mandible: A case report

**DOI:** 10.1016/j.ijscr.2023.108971

**Published:** 2023-10-24

**Authors:** Mhammad Ali, Mouhamad Hamama, Kheder Kheder, Orwa Haidar

**Affiliations:** a Al-Andalus University for Medical Science, Tartus, Syria; b Mesyaf National Hospital, Department of Oral and Maxillofacial Surgery, Syria

**Keywords:** Central giant cell granuloma, Mandible, Oral and maxillofacial surgery, Case report

## Abstract

**Introduction:**

Central Giant Cell Granuloma (CGCG) is a non-neoplastic benign lesion. It is primarily observed in the maxilla and mandible, with the mandible being the more reported site of the lesion. The lesion often manifests in the anterior regions of the mandible, extending occasionally across the midline. This case reports a rare presentation in the posterior portion of the mandible, in an edentulous area.

**Presentation of case:**

A 33-year-old female with a history of extraction of teeth 36 and 37 six months ago presented with a main complaint of a mass in the oral cavity. The oral examination revealed an expansive multilocular mass (4 × 3 cm) located on the alveolar ridge in the left posterior portion of the mandible, extending around tooth 33 with an intact masseter muscle. The histopathological findings were consistent with CGCG. Consequently, the lesion was surgically removed with no clinical or radiological recurrence observed during the 4-month post-operative follow-up.

**Discussion:**

While previous reports of CGCG in the posterior portion of the jaw showed destructive lesions that caused mandibular ramus destruction along with swollen masseter muscle, this case reports no involvement of the masseter muscle. Also, while some studies linked CGCG to tooth-bearing regions, our case suggests a possible traumatic link even after extraction.

**Conclusions:**

This case presents a rare CGCG occurrence in the posterior jaw, notably without masseter muscle involvement. It also indicates that CGCG can manifest in edentulous regions.

## Highlights

• This case represents a rare presentation of CGCG in the posterior portion of the mandible.
• This CGCG case reports no involvement of masseter muscle.
• This case suggests that CGCG can manifest even in edentulous areas.

## 1 Introduction

Central Giant Cell Granuloma (CGCG) is a non-neoplastic benign process, with the majority of cases (75 %) emerging before the age of 30, predominantly affecting children and young adults. Notably, it exhibits a higher incidence in females, occurring at a ratio of 2 to 1 compared to males [1].

CGCG is primarily observed in the maxilla and mandible. Interestingly, it is less frequently seen in the mandible than in the maxilla. These lesions often manifest in the anterior regions of the jaws, extending occasionally across the midline. In rare instances, they may affect the posterior jaws, including the mandibular ramus and condyle [1]. The anterior region of mandible is more frequently involved [2]. CGCG also can be confined to the tooth-bearing areas of the jaws [3]. 80 % of CGCG cases are involving the region anterior to the first premolar and rarely noticed in the posterior segment [4]. The differential diagnosis of CGCG includes aneurysmal bone cyst, benign chondroblastoma, brown tumor of hyperparathyroidism, cherubism, fibrous dysplasia, non-osteogenic fibroma, osteosarcoma and true giant cell tumor [5]. Fibrous dysplasia and other odontogenic tumors and non-odontogenic tumors can be easily ruling out on the basis of their clinical and radiological features and histopathology [6]. Brown cell tumor of hyperthyroidism usually occurs later in the life and is characterized by multiple lesions. Parathyroid hormone, serum and urinary levels of calcium, phosphate and bone or serum alkaline phosphatase are used in the diagnosis of brown cell tumors [6].

Treatments CGCG are: excision biopsies, curettage with safety margins, and partial or full resection of the affected bone; injecting corticosteroids into the affected region has been successful [7]. The most common treatment for CGCG of the jaws is curettage, especially for small, less aggressive lesions. However, recurrence is noted in approximately 20 % of cases [8]. Recurrent lesions may be managed by further curettage with peripheral ostectomy or by local resection. A variety of additional treatment modalities have been used for larger, more aggressive CGCG in an attempt to minimize surgical morbidity However, the success rates were inconsistent [8]. This case reports a rare presentation of CGCG in the posterior portion of the mandible in an edentulous area. This case has been reported in line with the SCARE Guidelines 2020 [9].

## 2 Presentation of case

**Fig. 1:**
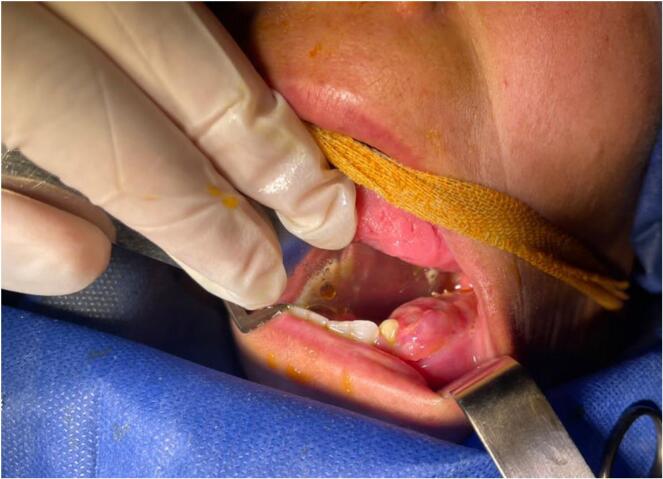
Intraoral features with multilocular mass on the alveolar ridge extended around the tooth 33.

A 33-year-old female patient complaining of mass in the oral cavity was referred by her general dentist to the oral and maxillofacial surgery department of National Hospital in Mesyaf, Hamah in March 2023. Her main complaint was a painless swelling lump in the left jaw that had grown rapidly and increased in size over the previous months. It was first noticed after extracting the teeth 36 and 37 six months ago, the teeth 34 and 35 were previously extracted without any following complications. The patient reported no severe infection has been in the extraction area or in tooth socket of the extracted teeth or even in the tooth 33. The patient reported no tingling sensation of the left lower lip or sensory disturbance of the left chin with disocclusion and difficulty speaking and chewing. The functional examination of mastication muscles revealed there is no harming, destruction or pain but light debility. The examination involved the masseter muscle - The closest muscle to lesion - which was intact. An intraoral examination revealed an expansive multilocular mass (4 × 3 cm) on the alveolar ridge in the left posterior portion of the mandible and it extended around the tooth 33 [Fig. 1]. The external features showed swollen gingivae above the jawline in the left side of mandible.

**Fig. 2:**
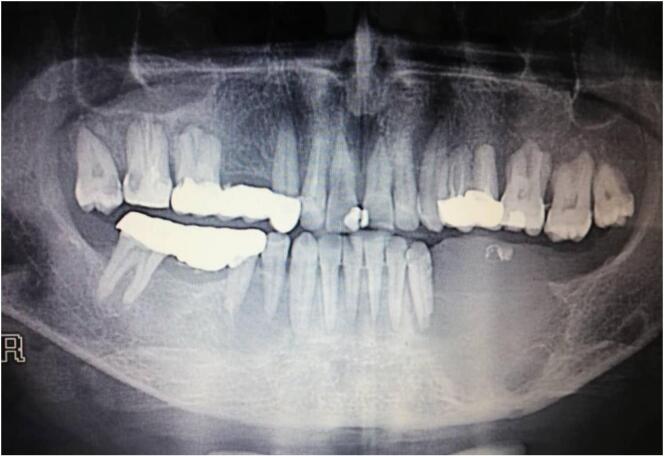
Huge corticated radiolucency with well-defined borders measuring 3 × 4 cm in panoramic radiograph.

The panoramic radiograph revealed a huge corticated radiolucency with well-defined borders of the left mandibular body measuring approximately 3 × 4 cm with minimum displacement of the teeth 33 [Fig. 2]. It involved the entire alveolar bone extending between the distal surface of tooth 32 and the tooth 37 extraction place (the tooth 33 is involved) crossing the alveolar margin of mandible which causes the difficulty in eating and chewing. The radiograph also showed a fine granular bone pattern around the lower border of the lesion.

**Fig. 3:**
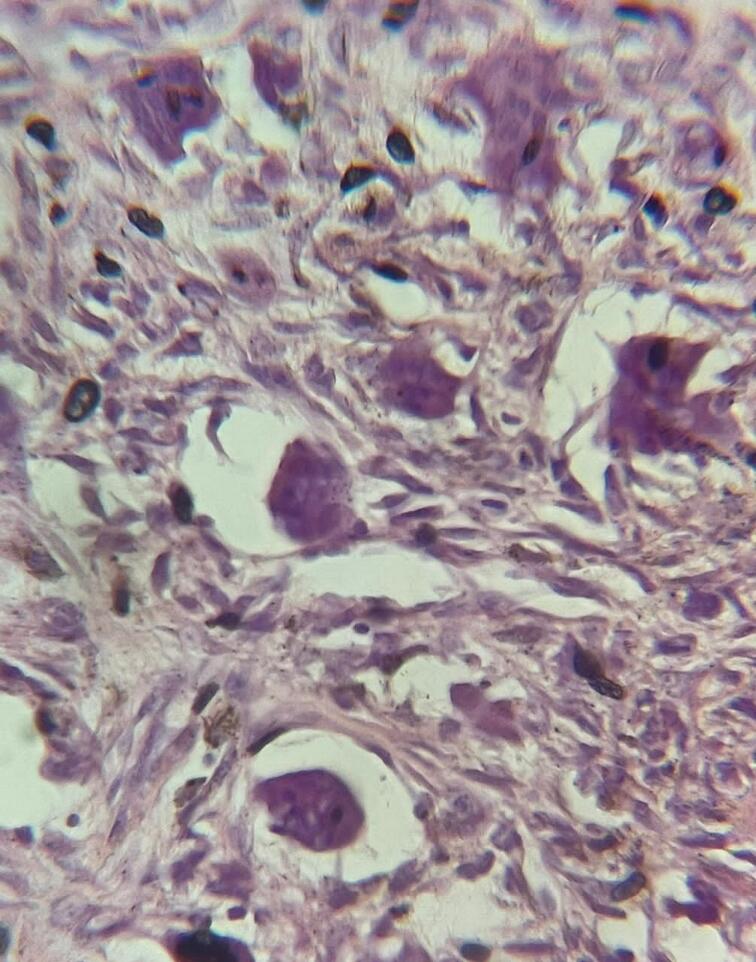
Aggregates of multinucleated giant cells and granulations tissues dispersed among stromal cells.

Histopathological department received fragments of fibrous mass measuring 4 × 3 cm; showing a soft white-gray tissue. Microscopic examination of serial sections revealed a neoplastic proliferation consisting of sheets of rounded to polygonal cells with collagenized zones and uniformly numerous multinucleated giant cells. There were no abnormal mitoses or malignant changes [Fig. 3]. These features were consistent with the diagnosis of CGCG.

**Fig. 4:**
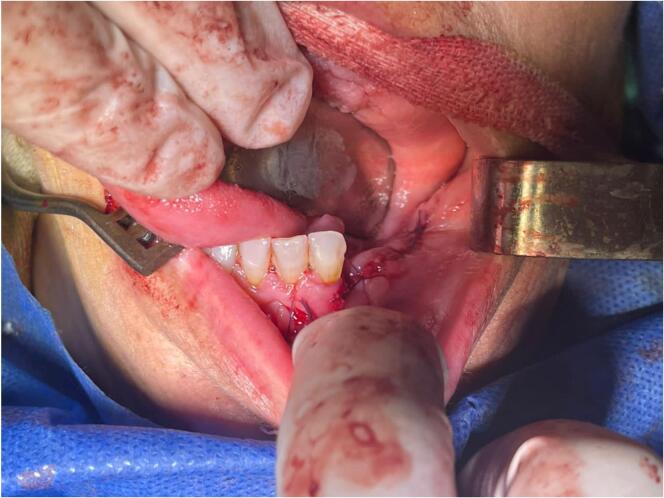
Intraoral operative features after the tumor was removed by curettage and en-bloc techniques.

**Fig. 5:**
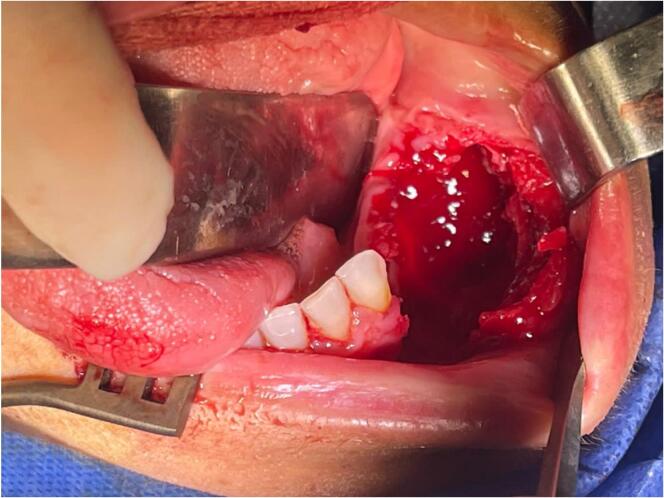
The tissues were closed in layers using vicryl (3.0).

Consequently, intraoral curettage including en-bloc resection was applied. Using intralesional steroids were excluded due to the acromegaly of the lesion, and the long period that intralesional steroids take to be effective in this case, in addition to the patient's continuous suffering if this treatment method applicated on long term. The lesion was removed en-bloc with a bony security margin of 1 cm to remove the peripheral margins of the lesion, extending into the alveolar bone to the lingual soft tissues. All the soft tissues involved in the lesion had to be removed, including tooth 33 [Fig. 4]. Following, the tissues were closed in layers using vicryl (3.0) [Fig. 5].

Face muscles functions were checked post-surgery. No facial nerves dysfunction was detected, the functional occlusion was treated without restrictions on movement or pain and mastication started returning back normal. No evidence of clinical and radiological recurrence was observed during post-operative follow-up for 4 months.

## 3 Discussion

The case we presented is a type of cases that reports a mandibular CGCG in the posterior portion, with swelling and pain as the main clinical features. CGCG is defined as an uncommon benign lesion of jaws that occurs in maxilla or mandible. Its clinical presentation is variable, ranging from an asymptomatic slow growing behavior to an aggressive painful lesion. Therefore, it is classified as a nonaggressive or an aggressive lesion depending on its radiological, pathological and clinical features [10]. The radiographs showed a deep opaque panicula and the pathological examination confirmed the CGCG case.

Previous studies demonstrated that many CGCG cases are located in the anterior portion of the jaw crossing the median line [11]. Notably, it is unusual to see this lesion in the posterior portion of the jaw. The cases that reported a CGCG of the posterior portion of the mandible showed relatively ill-defined, destructive lesions that caused mandibular ramus destruction along with swollen masseter muscle [12]. In contrast, our case reports a CGCG lesion of the posterior portion of the mandible with an intact masseter muscle without swelling. However, the patient had difficulty speaking and chewing as a result of the placement of the tumor and its large size.

According to the clinical and radiological examination, a differential diagnosis of fibrous dysplasia, aneurysmal bone cyst, non-osteogenic fibroma, osteosarcoma and brown tumor of hyperparathyroidism was made. Histologically, it is indistinguishable from other giant cell lesions of the bone like cherubis and aneurysmal bone cyst. Giant cell granuloma forms a lobulated mass of proliferative vascular forms a lobulated mass of proliferative vascular connective tissue packed with giant cells. These giant cells are seen lying in vascular stroma, these giant cells have a patchy distribution and signs of bleeding into the mass and deposits of hemosidrin are frequently seen [13].

One review illustrated that most CGCG patients developed the lesion without any signs of trauma, which suggests the neoplastic background of CGCG [14]. However, our patient developed the lesion in an edentulous region after 6 months of the extraction of a tooth without any reported side effects upon extraction. In contrast, one study reported that CGCG can be confined to the tooth-bearing areas of the jaws, meaning that it is related to the presence of teeth [15]. Donoff and Rosenberg discussed a case record of an uncomplicated extraction because of pericoronitis in the area of the lesion and claimed the local changes in the blood flow throughout the bone and local bone dysplasia could be probable etiologic factors [16]. Accordingly, our case supports the traumatic origin of CGCG and highlights the need to consider it even after tooth extraction. However, such an assumption requires further investigations and a large cohort. In our case, we can assume that teeth extractions was the CGCG inciting injury since the lesion was evident and grew rapidly only after molar teeth extractions.

Although the complicated localization of the CGCG in the posterior portion of the jaw in our case might draw attention to treat it conservatively, conservative treatments have yet not confirmed its priority over surgical treatment, especially in aggressive CGCG cases [17]. Accordingly, our patient underwent a successful surgical removal of the CGCG with no signs of recurrence on follow-up.

## 4 Conclusion

This case highlights a rare presentation of CGCG in the posterior portion of the jaw without involvement of the masseter muscle. It also indicates that CGCG can manifest in edentulous areas.

## Abbreviations

CGCG: Central Giant Cell Granuloma

## Informed consent

Written informed consent was obtained from the patient for publication of this case report and accompanying images. A copy of the written consent is available for review by the Editor-in-Chief of this journal on request.

## Ethical approval

Ethical approval is not applicable. The case report is not containing any personal information. The ethical approval is obligatory for research that involve human or animal experiments, so there is no institution that waived ethical approval.

## Funding

No source of funding.

## Author contribution

MA, MH: Drafting the article.

KK: Critical revision of the article.

OH: Supervision.

All authors provided final approval of the version to be submitted.

## Guarantor

Orwa Haidar _DDS, OMFS_.

## Conflict of interest statement

None.
